# Visceral leishmaniasis in acute myeloid leukemia revealed on peripheral blood smear

**DOI:** 10.1002/ccr3.1632

**Published:** 2018-06-04

**Authors:** Maxime Moniot, Maxime Loyens, Charles Mary, Coralie L’Ollivier

**Affiliations:** ^1^ CNRS, IRD, INSERM, AP‐HM, URMITE, IHU Méditerranée‐Infection Aix Marseille University Marseille France; ^2^ Department of Hematology and Vascular Biology CHU Conception AP‐HM Marseille France

**Keywords:** acute myeloid leukemia, amastigotes, kinetoplasts, *Leishmania infantum*, peripheral blood smear, visceral leishmaniasis

## Abstract

Images of parasitic forms of *Leishmania infantum* are typical in the hands of a skilled expert but should be known by biologists of Hematology Department. In an endemic region, the diagnosis of visceral leishmaniasis (VL) must be considered because of its potential role in accelerating hematological malignancy.

A 77‐year‐old male with myelodysplastic syndrome transformed into acute myeloid leukemia was hospitalized for weight loss and fever. At hospital admission, the blood work showed pancytopenia and inflammatory syndrome: hemoglobin 8.4 g/dL, leukocytes 0.7 × 10^9^/L, platelet count 6 × 10^9^/L, and C reactive protein 340 mg/L. Peripheral blood smear revealed neutrophils with intracellular microorganisms bearing prominent nuclei and adjacent rod‐shaped kinetoplasts (Figure 1 magnification x500; Figure 2 magnification x1000; wright stain). *Leishmania infantum* identification was confirmed by polymerase chain reaction assays targeting *L. infantum* kinetoplastic DNA. Retrospectively, visceral leishmaniasis (VL) diagnosis was confirmed by positive DNA detection for *L. infantum* on bone marrow smear, 2 months before finding amastigotes forms in peripheral blood. The patient subsequently died a few days later.

**Figure 1:**
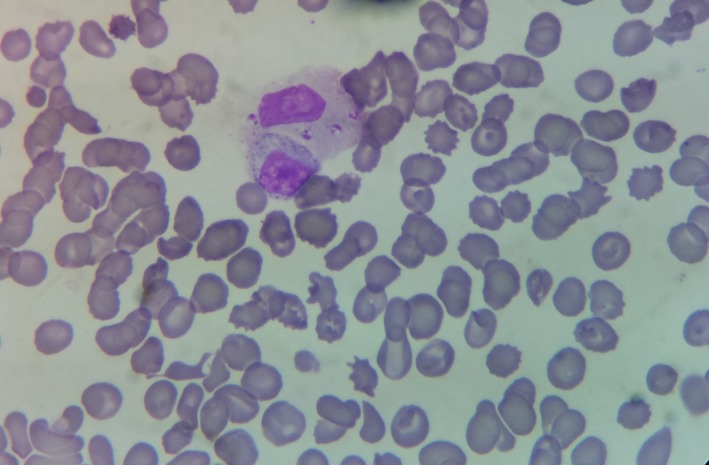
Amastigote forms of *Leishmania infantum* in white blood cells. Original magnification: x500; wright stain

**Figure 2:**
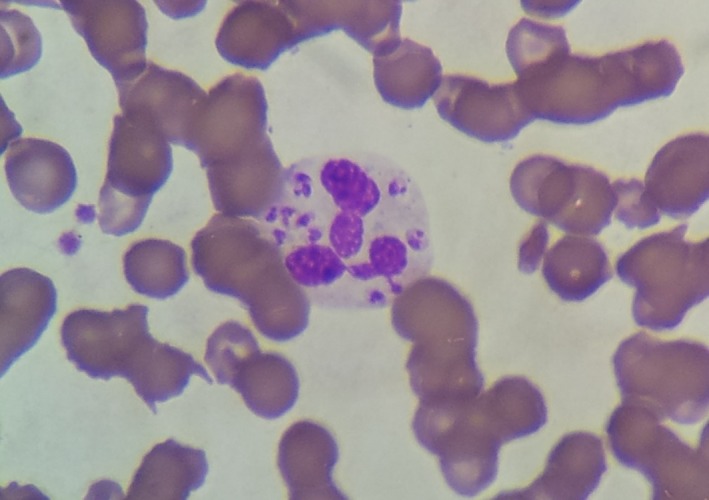
Amastigote forms of *Leishmania infantum* in white blood cells. Original magnification: x1000; wright stain

Hematological malignancies were reported as an underlying condition of VL but non‐HIV immunosuppressed patients are often misdiagnosed due to a frequent atypical presentation and overlap of typical signs.^1^ Microscopy diagnosis of VL requires samples collected by invasive means, such as bone marrow, whereas direct visualization of the amastigotes within peripheral monocytes or neutrophils is more unusual.^2^ The present case illustrates the importance of a vigilant examen of peripheral blood smear which can lead to fortuity‐diagnosed VL.

## CONFLICT OF INTEREST

None declared.

## AUTHORSHIP

MM: drafted the manuscript and obtained photographs of the organism. ML: contributed to patient care. CM: contributed to patient care and revised the manuscript. CL: contributed to patient care and revised the manuscript.
